# White Matter Microstructural Injury in Patients With Acute Single Subcortical Infarction: A Preliminary Tract‐Based Spatial Statistics With Atlas‐Based Analysis

**DOI:** 10.1155/np/1200067

**Published:** 2026-05-24

**Authors:** Zilong Zhu, Tianyi Shen, Junfeng Xiong, Tianrui Zhang, Zheng Sun, Hoang Thi Phong Lan, Chuanyou Li, Yang Ding, Jianbin Zhang

**Affiliations:** ^1^ Department of Acupuncture and Moxibustion, Second Affiliated Hospital of Nanjing University of Chinese Medicine, Nanjing, 210017, Jiangsu, China, njucm.edu.cn; ^2^ Department of Rehabilitation Medicine, First People’s Hospital of Changshu City, Changshu, 215500, Jiangsu, China; ^3^ Department of Medical Imaging, Second Affiliated Hospital of Nanjing University of Chinese Medicine, Nanjing, 210017, Jiangsu, China, njucm.edu.cn

**Keywords:** acute single subcortical infarction, Atlas-Based Analysis, Tract-Based Spatial Statistics, Wallerian degeneration

## Abstract

**Background:**

Acute single subcortical infarction (ASSI) is a prevalent cerebrovascular disorder in clinical practice. Although progress has been made in understanding its central mechanisms, the patterns of white matter (WM) microstructural damage in early‐stage unilateral lesions remain poorly characterized.

**Objective:**

This study aimed to examine WM microstructural alterations in ASSI patients using Tract‐Based Spatial Statistics (TBSS) and Atlas‐Based Analysis (ABA), and to assess the clinical utility of this combined approach.

**Methods:**

This prospective study enrolled 44 participants (20 patients with ASSI and 24 healthy control [HC] group). WM microstructure was evaluated using fractional anisotropy (FA), mean diffusivity (MD), axial diffusivity (AD), and radial diffusivity (RD). TBSS was employed for voxel‐wise analysis of the WM skeleton, while ABA was used to extract regions of interest (ROIs). Correlation analyses assessed associations between microstructural changes and Fugl‐Meyer Assessment (FMA) scores.

**Results:**

Relative to the HC group, patients with ASSI showed significantly reduced FA in multiple bilateral WM tracts (*p* < 0.05), including bilaterally in the corticospinal tract (CST), cingulate gyrus, hippocampus, and inferior fronto‐occipital fasciculus, as well as the right inferior longitudinal fasciculus (ILF), right superior longitudinal fasciculus, right superior longitudinal fasciculus (temporal part), and forceps major. ASSI patients also showed higher MD in several major WM tracts (*p* < 0.05), involving bilaterally in the anterior thalamic radiation and hippocampus, as well as the right CST, right cingulate gyrus, forceps major, right inferior fronto‐occipital fasciculus, right ILF, right superior longitudinal fasciculus, and right superior longitudinal fasciculus (temporal part). The changes in AD and RD values were not statistically significant. FMA scores are closely correlated with the MD and FA of the right cingulate gyrus. The MD of the right hippocampus and right ILF is also associated with FMA scores (*p* < 0.05).

**Conclusion:**

The combined TBSS‐ABA approach provides a reliable means to evaluate WM damage in ASSI. It not only identifies lesion clusters but also precisely localizes impaired WM regions, facilitating motor dysfunction assessment.

**Trial Registration:**

Chinese Clinical Trial Registry: ChiCTR2400085342

## 1. Introduction

Acute single subcortical infarction (ASSI) is a cerebrovascular disorder caused by occlusion of penetrating arteries, resulting in localized ischemia, hypoxia, and subsequent necrosis of brain tissue. This condition has high incidence and disability rates, significantly impairing patients’ quality of life and imposing substantial socioeconomic burdens [1].

White matter (WM) exhibits greater susceptibility to ischemic injury and typically sustains more extensive damage than gray matter [2, 3]. As a fundamental component of the central nervous system, WM forms the structural foundation of brain networks through interregional connectivity [4]. WM networks enable precise integration of multisensory inputs, including visual, proprioceptive, vestibular, and sensorimotor information—allowing subcortical regions to coordinate accurate movement planning and control [5]. WM damage constitutes a critical element of ischemic injury, with stroke‐induced damage causing persistent sensorimotor and cognitive impairments [6]. Motor dysfunction represents a frequent poststroke complication and primary focus of clinical rehabilitation. Consequently, characterizing poststroke WM microstructural damage provides crucial insights into infarction pathophysiology and facilitates functional assessment of motor deficits [7, 8].

Diffusion tensor imaging (DTI) technology, which is sensitive to microstructural tissue properties, enables in vivo investigation of WM anatomy and structure for both research and clinical applications [9]. With expanding applications and improved technical reliability, DTI promises significant value for treatment planning, preclinical marker detection, and microstructural pathology identification [10]. Although DTI studies have examined poststroke WM microstructural changes [11], limited evidence characterizes WM damage in early‐stage subcortical infarction.

Early WM investigations frequently utilized hand‐drawn regions of interest (ROIs) delineation for target region analysis [12, 13], potentially introducing subjective bias. Tract‐Based Spatial Statistics (TBSS) enhances sensitivity, objectivity, and interpretability for multi‐subject diffusion analyses compared to hand‐drawn ROI methods [14, 15]. This technique obviates predefined ROI requirements. By aligning major WM tracts across subjects, it enables precise intergroup comparisons. The method controls family‐wise error (FWE), reducing false‐positive rates. Statistical implementation permits visualization of WM microstructural damage. TBSS detects WM damage characteristics and remodeling patterns in chronic stroke phases [11, 16]. However, TBSS’s heightened sensitivity correlates with reduced specificity. The method detects extensive WM damage voxels, revealing group‐difference clusters that typically span multiple brain regions. Even under simulated conditions, TBSS may confound adjacent tracts [14]. Therefore, complementary ROI‐based approaches remain essential [17].

Atlas‐Based Analysis (ABA) is a type of ROI analysis that identifies individual brain WM structures based on the labels of a specific WM tractography atlas, rather than hand‐drawn methods [9]. ABA delivers detailed regional WM characterization for targeted neuroanatomical analysis [18, 19].

Therefore, this study employs the combined analysis of TBSS and ABA to investigate changes in the microstructure of WM in patients with ASSI and explore their relationship with motor function, thereby providing a reference for more precise diagnosis and assessment of patients with subcortical infarction.

## 2. Materials and Methods

### 2.1. Study Design

This single‐center study was conducted from June 2024 to October 2024. It was approved by the Ethics Committee of the Second Affiliated Hospital of Nanjing University of Chinese Medicine (2024SEZ‐003‐01) and adhered to the principles of the Declaration of Helsinki. Written informed consent was obtained from all participants.

### 2.2. Participants

The study consisted of 25 enrolled patients with ASSI in the ASSI group. The inclusion criteria included: (1) Diagnostic criteria for acute ischemic stroke with hemiparetic motor dysfunction; (2) Lesions located in the right subcortex; (3) Ages ranging from 40 to 80 years, no gender restrictions, right‐handedness; (4) Medical Research Council (MRC) muscle strength grade <4; (5) MRI acquisition ≤72 h postonset. The exclusion criteria included: (1) previous history of stroke; (2) participants with neurological dysfunctions due to other diseases such as brain tumors and cerebral parasitic diseases; (3) those with combined functional abnormalities of organs such as heart, lungs, liver, and kidneys or other severe diseases in the acute phase; (4) those unable to complete MRI (such as those with implanted cardiac pacemakers, metallic implants in the body, or those who are emotionally restless); (5) any brain abnormalities except infarction identified by MRI. The study enrolled 28 participants in the healthy control (HC) group. The HC group consisted of individuals aged 40–80 years old who were right‐handed and did not have a diagnosis of an acute infarct, TIA, or history of stroke (ischemic or hemorrhagic) or TIA. Diagnosis was made by a board‐certified vascular neurologist in each case. It was confirmed that participants did not have demyelinating disease or a prior stroke diagnosis before inclusion in this study.

### 2.3. MRI Data Acquisition

MRI data were acquired using an MRI scanner (MAGNETOM Vida3.0T, Siemens) equipped with a 32‐channel head coil. Identical protocols were applied to ASSI patients and HC group, including: 3D T1‐weighted (T1WI), T2‐weighted (T2WI), diffusion‐weighted imaging (DWI), fluid‐attenuated inversion recovery (FLAIR), and DTI.

Three‐dimensional T1‐weighted imaging data were acquired with a fast spoiled gradient echo sequence: field of view = 250 × 250, repetition time = 1900 ms, echo time = 3.82 ms, slice thickness = 1 mm, no slice spacing, voxel size = 1 × 1 × 1 mm^3^, matrix = 256 × 256.

Diffusion MRI data were acquired with a single‐shot echo‐planar imaging sequence: field of view = 256 × 256 mm^2^, repetition time = 10,000 ms, echo time = 90 ms, slice thickness = 2.0 mm, no slice spacing, number of slices = 74, voxel size = 2 × 2 × 2 mm^3^, matrix = 128 × 128, 30 gradient directions at *b* = 1000 s/mm^2^, one unweighted image at *b* = 0. A board‐certified neuroradiologist performed visual quality control on all datasets prior to analysis.

### 2.4. MRI Data Processing

DTI data processing was performed using the FMRIB Software Library (FSL, Version 5.0.9; http://www.fmrib.ox.ac.uk/fsl). The dcm2niigui tool was used to convert DICOM data into NIfTI data. The preprocessing of DTI data involved the following steps: the Brain Extraction Tool (BET) was used to remove nonbrain tissue, and the EDDY tool was employed for further preprocessing, including correction for inhomogeneity, head motion, and eddy current distortions [20]. Then, fractional anisotropy (FA), mean diffusivity (MD), radial diffusivity (RD), and axial diffusivity (AD) values were calculated by DTIFIT of FSL.

Using the TBSS toolkit within FSL [21], the FA images were nonlinearly registered across groups. After registration, the FA images were averaged to create a mean FA skeleton, which was then used to extract the characteristic values of different brain regions based on the JHU WM tractography atlas. The JHU‐ICBM atlas probabilistically defines 20 WM tracts through deterministic tractography averaging [22]. Skeletonization extended to AD, RD, and MD metrics, with FA‐based spatial normalization to the WM skeleton. Skeletonized data underwent statistical testing.

Preprocessed FA and MD values were registered to the MNI (Montreal Neurological Institute) standard space using individual T1‐weighted images. After obtaining individual ROIs in the diffusion space based on the JHU WM tractography atlas, the DTI metrics for the 20 brain regions were calculated using the fslmaths command.

### 2.5. Clinical Assessments

The Fugl‐Meyer Assessment (FMA) Scale, with a total score of 100 points, was used to evaluate the severity of motor dysfunction. Lower scores indicate more severe motor impairment.

### 2.6. Statistical Analysis

Demographic, clinical, and neuropsychological data were analyzed using SPSS Statistics 22. Continuous variables were assessed with Student’s *t*‐tests, while categorical variables were evaluated with chi‐square tests. Group differences in diffusion metrics (FA, MD, AD, and RD) were evaluated using nonparametric permutation testing in FSL’s randomise tool. Voxel‐wise statistical analysis of diffusion metrics across the WM skeleton used 5000 permutations in ASSI patients. Multiple comparisons were corrected using FWE control with threshold‐free cluster enhancement (TFCE) in FSL’s randomise tool [23, 24]. Parameter values from 20 brain regions defined by the JHU WM tractography atlas were correlated with FMA scores. Pearson’s correlation was used for normally distributed data; otherwise, Spearman’s correlation was employed. All tests were two‐tailed, with statistical significance thresholded at *p* < 0.05.

## 3. Results

### 3.1. Clinical and Demographic Data

**Figure 1:**
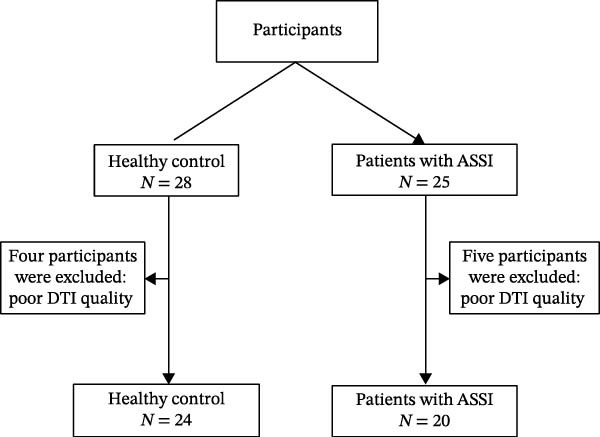
Flowchart of the participant inclusion and exclusion process.

As shown in Figure 1, 53 participants were initially enrolled in this study. After excluding data of insufficient quality, 20 patients with ASSI (8 men and 12 women, age range 41–77 years, with an average age of 63.65 ± 8.98) and 24 HC participants (8 men and 16 women, aged 42–77 years, with an average age of 62.33 ± 8.92) were finally included in the study. There were no statistical differences in these baseline characteristics between the ASSI and HC groups. Participant demographics and clinical characteristics appear in Table 1.

**Table 1:** Participants’ characteristics.

Demographic variables	ASSI group (*N* = 20)	HC group (*N* = 24)	*p*‐Value
Gender (M/F)	8/12	8/16	0.76
Age (years)	63.65 ± 8.98	62.33 ± 8.92	0.63
Disease duration (hours)	31.05 ± 15.23	–	–
Hypertension	12 (60%)	9 (37.5%)	0.14
Diabetes mellitus	6 (30%)	4 (16.7%)	0.49
Smoking	8 (40%)	7 (29.2%)	0.66
Alcohol intake	7 (35%)	3 (12.5%)	0.16
FMA	63.90 ± 27.09	–	–

Abbreviations: ASSI, acute single subcortical infarction; F, female; FMA, Fugl‐Meyer Assessment; HC, healthy control; M, male.

### 3.2. TBSS Results for FA Maps

**Figure 2:**
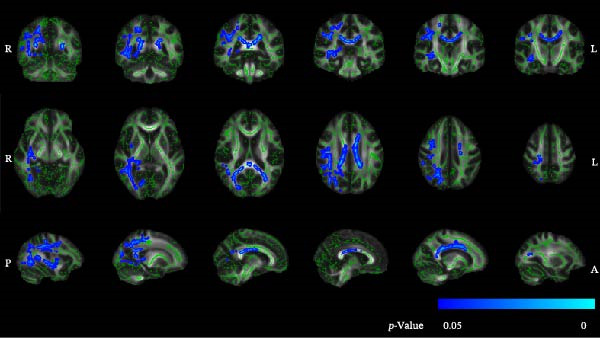
Differences in FA between groups (TBSS results). Green represents the anatomy of white matter tracts. Blue pixels indicate regions where significantly decreased FA values were observed in ASSI patients compared to the HC group.

Compared to HC, ASSI patients showed significantly reduced FA values in multiple WM regions (Figure 2). The main changes were found bilaterally in the corticospinal tract (CST), cingulate gyrus, hippocampus, and inferior fronto‐occipital fasciculus, as well as the right inferior longitudinal fasciculus (ILF), right superior longitudinal fasciculus, right superior longitudinal fasciculus (temporal part), and forceps major. In terms of FA values, the regions showing significant differences (*p* < 0.05) between the two groups are depicted in Table 2.

**Table 2:** White matter tracts in patients with ASSI show reduced FA values compared to the HC group.

Cluster	Voxels	MNI coordinates			Tracts that each cluster belongs to	*p*‐Value
		*X*	*Y*	*Z*		
1	83	28	−85	−4	Inferior fronto‐occipital fasciculus R Inferior longitudinal fasciculus R	0.049
2	2263	35	−39	31	Inferior longitudinal fasciculus R Superior longitudinal fasciculus R Superior longitudinal fasciculus (temporal part) R	0.037
3	2709	38	−45	0	Forceps major Inferior fronto‐occipital fasciculus R Inferior longitudinal fasciculus R Superior longitudinal fasciculus R Superior longitudinal fasciculus (temporal part) R	0.040
4	4105	−17	−33	29	Corticospinal tract L Corticospinal tract R Cingulate gyrus L Cingulate gyrus R Hippocampus L Hippocampus R Forceps major Inferior fronto‐occipital fasciculus L Inferior fronto‐occipital fasciculus R	0.036

Abbreviations: ASSI, acute single subcortical infarction; FA, fractional anisotropy; HC, healthy control; L, left; MNI, Montreal Neurological Institute; R, right.

### 3.3. TBSS Results for MD Maps

**Figure 3:**
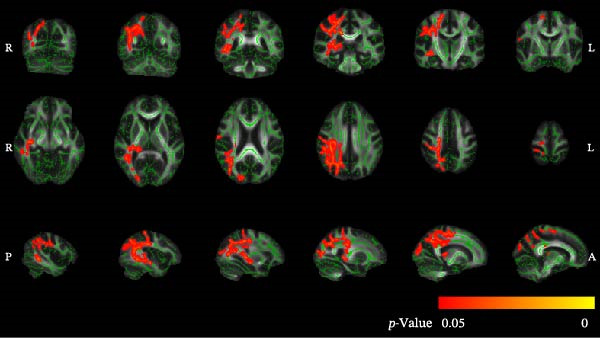
Differences in MD between groups (TBSS results). Green represents the anatomy of white matter tracts. Orange‐red indicates regions where significantly increased MD values were observed in ASSI patients compared to the HC group.

Patients with ASSI exhibited significantly higher MD values in several regions of WM compared to the HC group (Figure 3). Significant changes were observed bilaterally in the anterior thalamic radiation and hippocampus, as well as in the right CST, right cingulate gyrus, forceps major, right inferior fronto‐occipital fasciculus, right ILF, right superior longitudinal fasciculus, and the right superior longitudinal fasciculus (temporal part). Significant differences in MD values (*p* < 0.05) between the two groups are shown in Table 3.

**Table 3:** White matter tracts in patients with ASSI show increased MD values compared to the HC group.

Cluster	Voxels	MNI coordinates			Tracts that each cluster belongs to	*p*‐Value
		*X*	*Y*	*Z*		
1	7929	39	−57	35	Anterior thalamic radiation L Anterior thalamic radiation R Corticospinal tract R Cingulum (cingulate gyrus) R Cingulum (hippocampus) L Cingulum (hippocampus) R Forceps major Inferior fronto‐occipital fasciculus R Inferior longitudinal fasciculus R Superior longitudinal fasciculus R Superior longitudinal fasciculus (temporal part) R	0.026

Abbreviations: ASSI, acute single subcortical infarction; HC, healthy control; L, left; MD, mean diffusivity; MNI, Montreal Neurological Institute; R, right.

### 3.4. TBSS Results for AD and RD Maps

No statistically significant differences were found between ASSI and the HC group in the AD and RD values (no clusters with *p* < 0.05 in all cases).

### 3.5. Associations Between WM Changes and Fugl‐Meyer in Patients With ASSI

**Figure 4:**
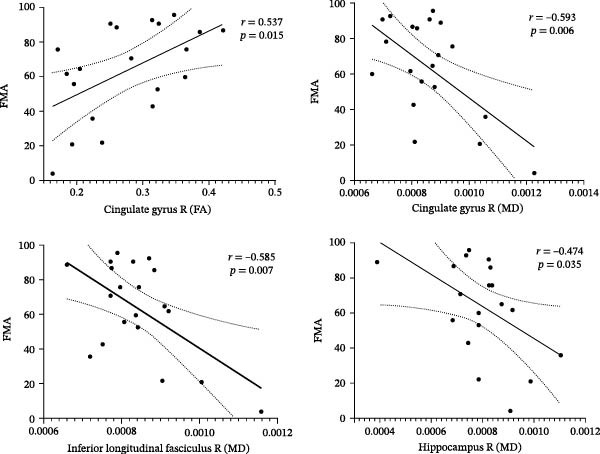
The correlation between characteristic values of relevant brain regions and motor function.

In ASSI patients, FMA scores significantly correlate with MD and FA values in the right cingulate gyrus. Additionally, MD values in the right hippocampus and right ILF also correlate with FMA scores (Figure 4). No significant correlations were observed in other brain regions. Full results are provided in Table S1.

## 4. Discussion

Subcortical infarction, a prevalent cerebrovascular disorder, often causes WM damage resulting in functional impairments. However, the characteristics of WM damage in acute unilateral subcortical infarction remain unclear. TBSS, an automated whole‐brain analysis method, detects voxel‐wise group differences by skeletonizing registered FA maps, thereby enhancing statistical power and visualizing WM microstructural damage. ABA extracts region‐specific WM metrics from these clusters using standardized atlases. This study employed TBSS combined with ABA to investigate WM microstructural alterations in ASSI patients. Our results demonstrate bilateral WM damage in ASSI patients. Furthermore, the study discovered a strong correlation between motor function and the right cingulate gyrus’s MD and FA values, as well as the MD values of the right hippocampus and the right ILF.

During the acute phase, most regions exhibiting decreased FA values in patients concurrently demonstrated increased MD values. FA describes the anisotropy of water molecule diffusion, representing the degree of tissue organization and alignment. MD describes the overall diffusive motion of water molecules [25]. When combined, these two parameters can accurately assess changes in the microstructure of WM. A decrease in FA values and an increase in MD values both indicate the destruction of WM fiber bundles [26]. Our findings demonstrate consistent FA and MD patterns in reflecting WM integrity. Nevertheless, it is noteworthy that the MD values of the anterior thalamic radiation on both sides of the patients were higher than those in the HC group. While higher MD values suggest less restriction on water molecules, such as inflammatory cell infiltration and cytotoxic edema (increased intracellular water) [27]. In the early stages of stroke (2–4 h) [28–31], MD in brain regions is mainly reduced, and then MD continues to rise, which is associated with Wallerian degeneration of brain tissue. Studies have shown that within 29 weeks after stroke, Wallerian degeneration is still the main issue, and the pyramidal tract is primarily affected. Animal studies have also found that WM fiber bundles far from the lesion site still exhibit ongoing damage up to 48 weeks after infarction, indicating that there may be a lag between changes in FA and MD [32]. No significant AD or RD abnormalities were observed. Previous studies have shown that RD values significantly increased from acute and subacute stages and from subacute to chronic stages, and AD values significantly increased from acute and subacute stages and from subacute to chronic stages [33]. In the early stages (within a few hours of onset), pathological changes in brain tissue such as edema and cellular swelling may not have fully developed yet, and there may not have been enough time to cause significant changes in AD and RD values, resulting in no significant abnormalities in AD and RD values [34]. Limited sample size and statistical power may represent additional contributing factors.

Our results revealed extensive WM microstructural damage postinfarction, affecting both ipsilateral and contralateral hemispheres. Studies have shown that WM is more susceptible to ischemic damage than gray matter [2, 3]. Early after ischemic stroke, there is an increase in vascular density around the lesion, accompanied by neuronal loss, aggregation, and activation of microglia [35, 36]. The loss of neurons and inflammation extend to other areas, leading to an expansion of the WM damage [37]. Acute ischemic edema may induce mild herniation, propagating ischemic injury to distant regions with consequent axonal damage [38]. At the same time, the corpus callosum, which is the largest bundle of WM fibers connecting the two cerebral hemispheres, is highly susceptible to damage in the early stages of ischemic stroke. This damage affects the connection and coordinated communication between the two hemispheres, leading to secondary destruction of the microstructure of WM in both hemispheres [39, 40].

The correlation analysis based on the ABA ROIs with motor function indicates that the MD and FA values of the cingulate gyrus on the side of the lesion, as well as the MD of the hippocampus and ILF at the side of the lesion, are closely related to the motor function of the patients. This suggests that after ASSI, the integrity of the cingulum and ILF at the site of the lesion is associated with the motor capacity of the patients. Studies have found that motor function is related to visual and spatial processes. During affected‐hand grasping tasks, motor‐impaired patients show bilateral cingulate gyrus activation [41]. Brain injury frequently induces bilateral cingulum damage with progressive secondary WM degeneration [42, 43]. The cingulum constitutes the principal fiber system connecting the cingulate gyrus, medial cortex, and medial temporal lobe to other brain regions. Additionally, the cingulum contributes to the default mode network (DMN) architecture [44, 45]. The DMN comprises interconnected regions showing high baseline activity during rest. Poststroke DMN abnormalities, including altered connectivity and motor regulation patterns, correlate with motor deficits [46–48]. The ILF is a major associative tract connecting temporal and occipital lobes, facilitating ipsilateral communication for visual processing, language comprehension, and emotion recognition. ILF structural integrity also correlates with motor function [49]. We found that ipsilesional ILF microstructural integrity relates to motor function. Previous studies have shown that ILF is associated with language and cognitive functions [50, 51], and it is rarely mentioned in studies on central structural damage in cerebral infarction. The ILF is a large associative WM bundle that connects the occipital cortex with the anterior temporal structures in a bidirectional manner. Given its cortical projection patterns and recently demonstrated multilayered anatomical organization, the ILF is considered crucial for maintaining functional processes in the visual modality, and the misprocessing of this visual information can severely affect the patient’s motor function [52].

Our study detected microstructural damage in bilateral CST and corpus callosum postinfarction. However, MD/FA values in these tracts showed no significant correlation with motor function, contrasting with previous reports [39, 53, 54]. The CST’s microstructural integrity typically serves as a primary biomarker for poststroke motor function, which strongly depends on lesion location and CST integrity [55]. The results of this study may be due to the widespread damage to the CST in early subcortical infarction patients, which is still in the progressive phase of Wallerian degeneration, and the insensitivity to motor function [56]. Other potential factors include unstratified infarction severity and limited sample size. Furthermore, early CST damage concentrates near lesions, and ROI‐averaged metrics may dilute correlations. Automated tract‐specific analysis could better elucidate clinical correlations [49].

Our study has several limitations. First, the sample size was relatively small, and the study design was cross‐sectional. Additionally, gender differences may introduce bias in the results. The analysis method based on DTI‐TBSS combined with ABA can visually assess the characteristics of damage through image display and perform quantitative calculations for each fiber bundle. Further clinical analysis is needed to examine the damage characteristics of different segments of each fiber tract. Cerebral infarction is a complex pathological process, and motor function is closely related to multiple functional brain regions. In the future, larger sample sizes and multicenter, multimodal longitudinal analyses are needed to provide insights into the characteristics of specific WM fiber bundle damage and the injury and reorganization features of brain networks after cerebral infarction.

## 5. Conclusion

Patients with ASSI exhibit extensive WM microstructural damage, and motor function impairment is closely related to WM microstructural integrity. The research method combining TBSS and ABA can not only visualize the brain regions with WM microstructural damage but also further analyze the specific regional feature values and their relationship with motor function.

## Author Contributions

Conceptualization: Zilong Zhu and Jianbin Zhang. Investigation: Zilong Zhu, Junfeng Xiong, Tianrui Zhang, Zheng Sun, Hoang Thi Phong Lan and Tianyi Shen. Software: Zilong Zhu and Tianyi Shen. Methodology: Zilong Zhu, Tianyi Shen, Zheng Sun and Yang Ding. Project administration: Zheng Sun, Chuanyou Li, Yang Ding and Jianbin Zhang. Supervision: Yang Ding and Jianbin Zhang. Writing – original draft: Zilong Zhu, Tianyi Shen and Jianbin Zhang. Writing – review and editing: Zilong Zhu, Zheng Sun, Tianyi Shen, Yang Ding and Jianbin Zhang.

## Funding

This work was supported by grants from the Jiangsu Provincial Research Institute of Chinese Medicine Schools Open Project (Grant JSZYLP2024036) and the Postgraduate Research and Practice Innovation Program of Jiangsu Province (Grant SJCX24_1016).

## Conflicts of Interest

The authors declare no conflicts of interest.

## Supporting Information

Additional supporting information can be found online in the Supporting Information section.

## Supporting information


**Supporting Information** Table S1: Associations between white matter changes and Fugl‐Meyer in patients.

## Data Availability

The data that support the findings of this study are available upon request from the corresponding author. The data are not publicly available due to privacy or ethical restrictions.
