# Supplementary material for: White Matter Microstructural Injury in Patients With Acute Single Subcortical Infarction: A Preliminary Tract‐Based Spatial Statistics With Atlas‐Based Analysis
Source: Neural Plast. 2026 May 24;2026:1200067. doi: 10.1155/np/1200067 (PMC13199682; doi:10.1155/np/1200067)
Supplement: Supplementary file 1 — Supporting Information Table S1: Associations between white matter changes and Fugl‐Meyer in patients. [file NP-2026-1200067-s001.docx]

| MD | *r* | *P*-value |
| --- | --- | --- |
| 1 Anterior.thalamic.radiation.L | -0.088 | 0.712 |
| 2 Anterior.thalamic.radiation.R | -0.379 | 0.100 |
| 3 Corticospinal.tract.L | 0.050 | 0.835 |
| 4 Corticospinal.tract.R | -0.190 | 0.423 |
| 5 Cingulum.(cingulate.gyrus).L | -0.171 | 0.472 |
| 6 Cingulum.(cingulate.gyrus).R | -0.593** | 0.006 |
| 7 Cingulum.(hippocampus).L | -0.046 | 0.846 |
| 8 Cingulum.(hippocampus).R | -0.474* | 0.035 |
| 9 Forceps.major | -0.260 | 0.268 |
| 10 Forceps.minor | 0.051 | 0.830 |
| 11 Inferior.fronto-occipital.fasciculus.L | 0.174 | 0.463 |
| 12 Inferior.fronto-occipital.fasciculus.R | -0.358 | 0.122 |
| 13 Inferior.longitudinal.fasciculus.L | 0.054 | 0.821 |
| 14 Inferior.longitudinal.fasciculus.R | -0.585** | 0.007 |
| 15 Superior.longitudinal.fasciculus.L | -0.194 | 0.413 |
| 16 Superior.longitudinal.fasciculus.R | -0.121 | 0.613 |
| 17 Uncinate.fasciculus.L | 0.006 | 0.980 |
| 18 Uncinate.fasciculus.R | -0.241 | 0.306 |
| 19 Superior.longitudinal.fasciculus.(temporal.part).L | -0.016 | 0.947 |
| 20 Superior.longitudinal.fasciculus.(temporal.part).R | -0.241 | 0.307 |
| FA |  |  |
| 1 Anterior.thalamic.radiation.L | -0.290 | 0.214 |
| 2 Anterior.thalamic.radiation.R | 0.273 | 0.243 |
| 3 Corticospinal.tract.L | 0.279 | 0.234 |
| 4 Corticospinal.tract.R | 0.117 | 0.624 |
| 5 Cingulum.(cingulate.gyrus).L | 0.359 | 0.120 |
| 6 Cingulum.(cingulate.gyrus).R | 0.537* | 0.015 |
| 7 Cingulum.(hippocampus).L | 0.336 | 0.148 |
| 8 Cingulum.(hippocampus).R | -0.096 | 0.688 |
| 9 Forceps.major | 0.117 | 0.622 |
| 10 Forceps.minor | -0.057 | 0.812 |
| 11 Inferior.fronto-occipital.fasciculus.L | -0.028 | 0.906 |
| 12 Inferior.fronto-occipital.fasciculus.R | 0.156 | 0.512 |
| 13 Inferior.longitudinal.fasciculus.L | -0.236 | 0.316 |
| 14 Inferior.longitudinal.fasciculus.R | 0.073 | 0.761 |
| 15 Superior.longitudinal.fasciculus.L | 0.029 | 0.905 |
| 16 Superior.longitudinal.fasciculus.R | 0.105 | 0.659 |
| 17 Uncinate.fasciculus.L | -0.153 | 0.520 |
| 18 Uncinate.fasciculus.R | 0.299 | 0.200 |
| 19 Superior.longitudinal.fasciculus.(temporal.part).L | 0.028 | 0.906 |
| 20 Superior.longitudinal.fasciculus.(temporal.part).R | 0.111 | 0.640 |

Supplementary Material 1: Associations between white matter changes and Fugl-Meyer in patients.

Abbreviations: MD, mean diffusivity; FA, fractional anisotropy; L, left; R, right; * *p*<0.05; ** *p*<0.01.
